# Fatigue and Recovery of Muscles for Pulling Tasks

**DOI:** 10.3390/ijerph192215159

**Published:** 2022-11-17

**Authors:** Cannan Yi, Huali Zuo, Caijun Zhao, Kai-Way Li, Hong Hu, Fan Tang, Tong Long

**Affiliations:** 1 School of Safety and Management Engineering, Hunan Institute of Technology, Hengyang 421102, China; 2 Department of Industrial Management, Chung Hua University, Hsinchu 30012, Taiwan

**Keywords:** manual materials handling task, pulling task, muscle fatigue, muscle fatigue recovery, musculoskeletal disorders (MSDs)

## Abstract

Manual materials handling (MMH) contributes to musculoskeletal disorders (MSDs) in the workplace. The development and recovery of muscle fatigue are essential in work/rest arrangements for MMH tasks. A pulling experiment, including a muscle fatigue test and a muscle fatigue recovery test, was conducted. In the muscle fatigue test, the participant performed a pulling task on a treadmill with a walking velocity of 1 km/h until they could no longer do so. The load was either 30 or 45 kg. The maximum endurance time (*MET*) was recorded. The pull strength (*PS*) of the participant both before and after the pulling task was measured. The subjective ratings of muscle fatigue after the pulling task were recorded. In the muscle fatigue recovery test, the participant took a rest after performing the pulling task. The participants reported their subjective ratings of muscle fatigue on the *CR-10* scale after taking a rest for a time period *t,* where *t* = 1, 2,…, 6 min. The *PS* of the participant was then measured again. It was found that the load significantly affected the *MET* for pulling tasks. The load was insignificant to the decrease of the *PS*, but was significant to the decrease rate (*PS* decrease per min) of the *PS*. The *PS* decrease rate for the 45 kg condition (30.8 ± 16.5 N/min) was significantly higher (*p* < 0.05) than that of the 30 kg condition (15.4 ± 5.5 N/min). The recovery time significantly affected the *PS* and *CR-10*. Two *MET* models were established to explore the development of muscle fatigue in pulling tasks. A *PS* model was constructed to describe the recovery of muscle force. A *CR-10* model was proposed to show the subjective ratings of recovery. These models are beneficial for determining the work/rest allowance for pulling tasks.

## 1. Introduction

Manual materials handling (MMH) is common in the workplace and in daily life [1]. Nowadays, although mechanical equipment can substitute manual operations, many MMH activities, such as lifting, pushing, pulling, carrying, and holding, are still required in many industry sectors. MMH is generally physically demanding. Prolonged working time, overexertion, awkward posture, and excessive load cause muscle fatigue and could lead to musculoskeletal disorders (MSDs). MMH is the most common cause of occupational fatigue and low back injury [2]. In the USA, MMH contributes to over half a million cases of MSDs [3]. According to the Health and Safety Executive (HSE) statistics [4], there were approximately 470,000 workers who suffered MSDs in 2020 and 2021 in the UK. This accounts for 28% of all work-related illnesses.

Many lifting/lowering and carrying tasks have been replaced by pushing and pulling to reduce the physical requirements of workers [5,6,7,8]. The literature indicates that one-third or more of the employees in the United States need to push and pull at work [9,10,11]. Most material handling aids, such as carts and trolleys, may be pushed or pulled. However, some of the material handling aids can only be pulled. A pallet jack is one the example. Pulling involves efforts from body parts, including the upper limbs, trunk, and lower limbs. When pulling, the waist, back, and arms are the most fatigued body segments [8,10,12]. Frequent fatigue of these body parts could lead to MSDs [13,14]. The literature has shown that workers performing pulling tasks suffered a higher prevalence of shoulder and lower back pain [14,15].

Pulling tasks involving both static [16,17,18,19,20,21,22] and dynamic [13,23,24,25,26,27] postures have been reported. The difference between those two approaches was whether the participants needed to walk when they were pulling. Muscle fatigue may be defined as the reduction in the maximal capacity to generate force or power output [28]. The development of muscle fatigue for pulling tasks was generally assessed by measuring the pull force, electromyography (EMG) signal of muscle groups, joint stress, or maximum endurance time (*MET*). The *MET* models are often constructed to predict the maximum time an operator can persist at a worksite [19,20,21,27]. They provide a way to determine the work allowance for workers.

Breaks are required to allow recovery for MMH tasks. Recovery of muscle fatigue is the regain of muscle strength [29]. Exploring the relationship between the recovery level of muscle strength and the recovery time periods is essential for job designers. Muscle fatigue recovery is often assessed by measuring muscle strength over a recovery time period [30,31,32,33]. Muscle strength models have been reported established [30,31,32,33]. Those models are also helpful in determining rest allowance for workers.

Subjective ratings provide an easy assessment tool for muscle fatigue. The relationship between these ratings and objective muscle strength data has been reported [33,34,35,36]. Models based on subjective data were adopted to assess muscle fatigue [35,36,37] and muscle fatigue recovery [33]. These models provided estimations of muscle fatigue levels over a certain period.

In our previous study [27], a pulling task with a walking velocity of 1 and 2 km/h was conducted. We obtained the *MET* model to describe the maximum time a puller can persist. The levels of muscle fatigue recovery for pulling tasks under walking conditions, however, have not been explored. When conducting the muscle fatigue recovery test, an endurance experiment was usually adopted to induce muscle fatigue [30,31,33]. Therefore, we designed a muscle fatigue and a muscle fatigue recovery test for pulling tasks in this study. The aim of the current study was to explore the development of muscle fatigue and muscle fatigue recovery for pulling tasks. The development and recovery of muscle fatigue were quantified by measuring the pull strength (*PS)*, *MET*, and *CR-10* scores. *MET* predictive models were constructed to describe the development of muscle fatigue. Both a *PS* model and a *CR-10* model were obtained to predict the recovery level of muscle fatigue.

## 2. Materials and Methods

### 2.1. Experiment

A pulling task was performed in the laboratory. The temperature was 20.0 (±1.4) °C, and the relative humidity was 64.5 (±15.7)%.

### 2.2. Participants

**Figure 1:**
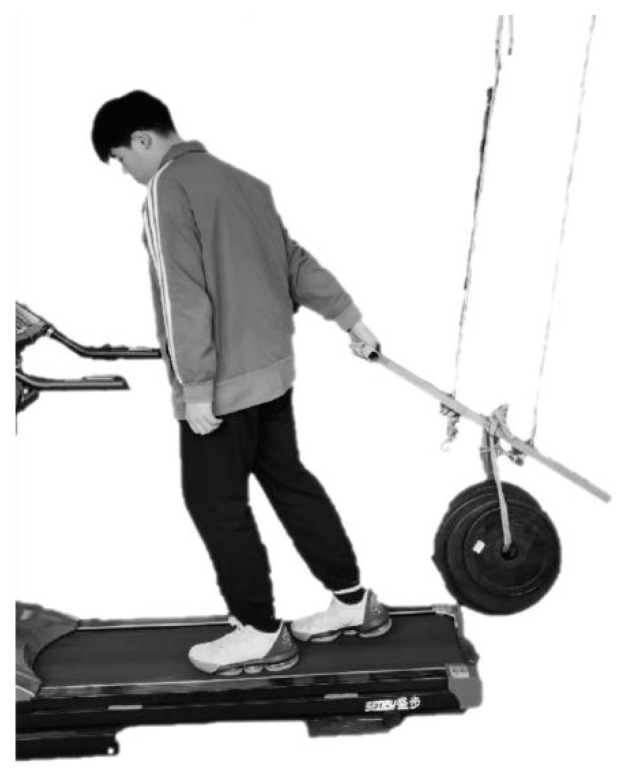
Pulling task on a treadmill.

**Figure 2:**
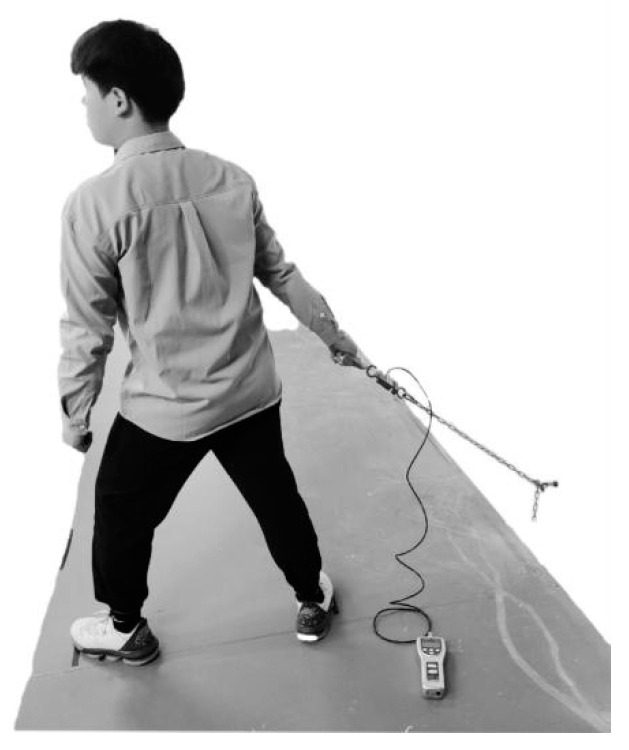
*PS* measurement.

Forty male adults participated in the study. Their age, body mass, stature, and body mass index (*BMI*) were 19.1 (±1.1) years, 65.9 (±10.4) kg, 168.8 (±3.1) cm, and 23.1 (±3.4) kg/m^2^, respectively. They were right-handed and healthy without a history of MSDs within a year of the study. All of them had no experience using a real pallet truck. Upon arriving in the laboratory, the participants were informed about the purpose and procedure of the experiment. They read and signed informed consent. Then, they were asked to pull and walk on a treadmill (see Figure 1) until they were familiar with pulling and walking. When measuring their *PS* (see Figure 2), they were instructed to pull in the same way as was performed on the treadmill, except they were not walking.

### 2.3. Apparatus and Tools

A stick-handle unit was fabricated to mimic the stick and handle of a real pallet truck. This unit contains a T bar (1.5 kg) that is 81.5 cm in length and has a handle with a diameter of 3 cm. It was hung to the ceiling using two steel wires. The lower end of the stick was approximately 37 cm over a treadmill platform. A weight (cast iron), of 30 or 45 kg, was hung at the middle of the stick to generate a backswing force when the handle was being pulled (see Figure 1). The forces required to pull these loads and maintain the posture in Figure 1 were approximately 77.2 N and 106.6 N, respectively. The trials of the two weights were performed in two different sessions and these sessions were at least 24 h apart.

A force sensor (Lutron^@^ Inc., FG-5100, Taipei, Taiwan) was installed to measure the *PS* (see Figure 2). It was connected to a chain. The lower end of the chain was linked with a hook 37 cm above the ground on the wall. There was a handle (ø3 cm) on the other side of the chain. When the participant pulled the handle (see Figure 2), a digital display showed the peak force of the pulling. The reading was the *PS*. Participants reported their subjective ratings of muscle fatigue on the *CR-10* scale [38] after the pull.

### 2.4. Experimental Design

Muscle fatigue was defined as a reduction in the ability to exert force in response to voluntary effort [39]. Reduction of muscle force [34,40], change of subjective ratings of muscle fatigue [41] before and after forceful exertion, and *MET* [20,42] for continuous forceful exertion have been used to assess muscle fatigue in the literature. This experiment included tests of muscle fatigue and muscle fatigue recovery by measuring the *MET*, *PS*, and *CR-10* scores.

In the muscle fatigue test, the participant pulled and walked on a treadmill at a velocity of 1 km/h (see Figure 1) until he could not continue any longer. The walking speed was determined following the recommendations in the literature [6,43,44,45,46] and the results of our pilot trials. Both the *MET* and *PS* before and after the test were recorded. The *PS* before the test was the maximum voluntary contraction (*MVC*) and the *PS* after the pull was recorded as *PS*_0_. After the trial, a *CR-10* rating [38] was recorded and was denoted as *CR-10*_0_.

In the muscle fatigue recovery test, participants took a rest for 6 min, and their *PS* and *CR-10* at the end of 1, 2, 3, 4, 5, and 6 min were measured. These *PS* and *CR-10* measures were termed *PS_i_* and *CR-10_i_*, respectively, for *i* = 1, 2, …, and 6 min.

### 2.5. Procedures

**Figure 3:**
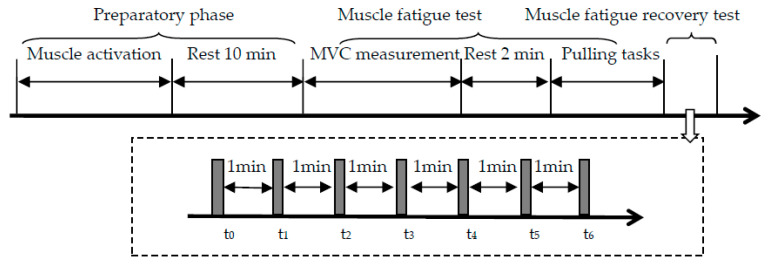
Experiment procedure.

Figure 3 shows the procedure of the experiment. In the preparatory phase, the participant performed a warmed-up exercise for 5 min to activate the muscles [47]. After a rest of 10 min, he performed the muscle fatigue test. In the muscle fatigue test, the participant completed the *MVC* measurement and then performed the pulling tasks. He pulled the handle with maximum effort for 4–6 s and the peak value of the pulling strength was denoted as *PS*. The *PS* was measured three times with an interval of 2 min between each measurement [48]. The maximum *PS* of these three was recorded as the *MVC*. After a rest of 2 min, the participant pulled and walked on a treadmill until he could not continue any longer. The time of his walking was his *MET*. The beginning of the muscle fatigue recovery test started at the end of the treadmill walking. This time was denoted as *t*_0_. The total recovery time was 6 min. At time *t_i_* (*i* = 0, 1, …, 6), the participant reported his *CR-10_i_* and his *PS_i_* was measured.

### 2.6. Data Processing

Descriptive statistics were conducted to show the *MET*, *PS*, and *CR-10* under experiment conditions. Analyses of variance (ANOVA) were performed to determine the effects of load on the dependent variables. The Duncan post hoc tests were conducted for posterior comparisons. Correlation analysis was performed to show the relationship among measured parameters. Regression analyses were carried out to establish the *MET*, *PS*, and *CR-10* prediction models. Microsoft^®^ Excel (Microsoft, Redmond, Washington, DC, USA) was used for preliminary data processing. The SAS^®^ 9.0 (SAS Institute Inc., Cary, NC, USA) was used for statistical analysis. A significance level of α = 0.05 was used.

## 3. Results

The ANOVA results showed that load significantly affected *MET* (*F* = 54.7, *p* < 0.0001), but was insignificant to *PS_i_* (*i* = 0, 1, …, 6) or *CR-10_i_* (*i* = 0, 1, …, 6) (*p* > 0.05). The mean *MET* of the 30 kg condition (14.0 ± 4.9 min) was significantly higher than that of the 45 kg condition (7.0 ± 3.5 min). The *PS* and *CR-10* were shown in Table 1. Both the *PS* and *CR-10* varied with recovery time (*p* < 0.0001). The Duncan results showed that *PS*_0_ was significantly smaller than others. *PS*_1_ was significant smaller than *PS*_3_, *PS*_4_, *PS*_5_, and *PS*_6_. *PS*_2_ was significantly smaller than *PS*_4_, *PS*_5_, and *PS*_6_. *PS*_3_ was significantly smaller than *PS*_5_ and *PS*_6_. *PS*_4_ was significantly smaller than *PS*_6_. The *CR-10*, on the other hand, became smaller as the recovery time increased. The *PS* and *CR-10* were significantly correlated (*r* = −0.45, *p* < 0.0001).

### 3.1. Muscle Fatigue for Pulling Tasks

**Figure 4:**
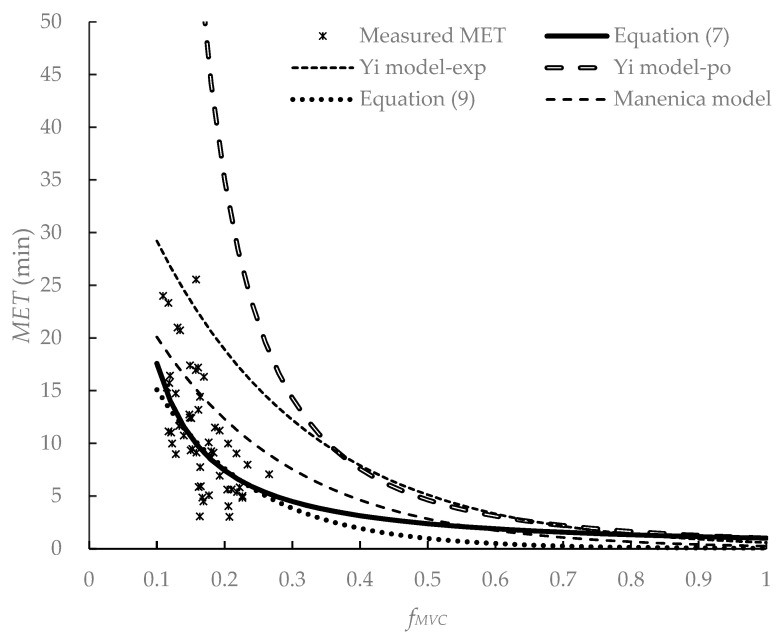
*MET* predictive models of muscle fatigue. Yi model-exp: *MET* model of the exponential function in Yi et al.; Yi model-po: MET model of the power function in Yi et al.; *f_MVC_* = forceful exertion/*MVC*.

In the literature, the *MET* models were often established using the relative load *f_MVC_* (*f_MVC_* = forceful exertion/*MVC*) or *%MVC* (*%MVC* = *f_MVC_* × 100) as the independent variable [21,39,49,50]. In dynamic force exertion tasks, such as carrying and pulling tasks, the development of muscle fatigue was usually evaluated by walking velocity, *f_MVC_*, and *BMI* [27,45,50,51]. By summarizing the *MET* models for static and dynamic force exertion, it was found that the *MET* is inversely proportionate with the *f_MVC_* in a curvilinear pattern. A nonlinear curve can be found when plotting the *MET*–*f_MVC_* relation. Observing the relationship between the *MET* and *f_MVC_* in this experiment (see Figure 4), it was found that the *MET* was significantly negatively correlated with *f_MVC_* (*r* = −0.62, *p* < 0.0001). Hagberg [50] found that there was no significant difference in the endurance time in static and slow dynamic elbow flexion. We hypothesized that the *MET* models based on static force exertion were suitable for slow walking tasks. In the current study, the participants pulled and walked with a velocity of 1 km/h. This walking velocity was slower than those in the literature [43,44]. We assumed that walking at a low velocity and pulling was a slow dynamic task and attempted to construct the *MET* muscle fatigue model for pulling tasks. The participants were split into two groups: participants No. 1–20 were in group A and participants No. 21–40 in were group B. Data in group A were used to construct models, while data in group B were used for model validation.

#### 3.1.1. Models According to Static Force Exertion

A summary of existing static *MET* models revealed [21,39,49,50] that exponential and power functions have been adopted to fit the *MET*–*f_MVC_* relation. Similar to those *MET* models in the literature [21,39,49,50], the *MET* for pulling tasks may be fitted using these two mathematic functions:

(1) $$MET=k\times e^{f_{MVC}^{}\times c}$$

(2) $$MET=k\times f_{MVC}^{c}$$

In Equations (1) and (2), both *k* and *c* are constant values. By logarithmic transformation of Equations (1) and (2), Equations (3) and (4) were obtained, respectively.

LN(*MET*) = LN(*k*) + *c* × *f_MVC_* (3)

LN(*MET*) = LN(*k*) + *c* × LN(*f_MVC_*) (4)

By simple intercept-free linear regression, we obtained:

(5) $$\mathrm{LN}(MET)=12.664\times f_{MVC}\;\;\;\;\;\;(p\;<\;0.0001,\;F\;=\;230.6,\;R^{2}\;=\;0.85)$$

(6) $$\mathrm{LN}(MET)=-1.245\times LN(f_{MVC})\;\;\;\;\;\;(p\;<\;0.0001,\;F\;=\;1223.6,\;R^{2}\;=\;0.97)$$

The model with the highest *R*^2^ (0.97) was selected. This model was rewritten as Equation (7).

(7) $$MET=f_{MVC}^{-1.245}$$

To determine the prediction error of the prediction models, the mean absolute deviation (*MAD*) was usually used in the literature [20,33,34,52]. The *MAD* was calculated using Equation (8):

(8) $$MAD=\frac{1}{n}\sum_{i=1}^{n}\left|measured\;value-predicted\;value\right|$$

By substituting data groups A and B into the *MET* Equation (7), the *MADs* were 3.28 (±2.99) min and 3.22 (±2.88) min, respectively, for these groups (see Table 2).

#### 3.1.2. Models of Dynamic Force Exertion

Similar to the *MET* models for dynamic force exertion in the literature [27,45,50,51], we had:

(9) $$MET=e^{-6.863f_{MVC}}\times BMI^{1.083}\;\;\;\;\;\;(p\;<\;0.0001,\;F\;=\;497.02,\;R^{2}\;=\;0.96)$$

*BMI* is in kg/m^2^. By substituting data groups A and B into Equation (9), the *MADs* were 3.57 (±3.08) min and 3.70 (±3.74) min, respectively, for these groups (see Table 2).

#### 3.1.3. Model Comparison

Yi et al. [19] proposed *MET* models of exponential function and power function for static pulling tasks. They found that Manenica’s body pull model [53] could better fit the *MET–f_MVC_* relation for static pulling tasks. We substituted our group B data into those three models. The *MADs* were much larger than those in the current study (see Table 2). We plotted those five models, including two models constructed by Yi et al. [19], a body pull model by Manenica [53], and the *MET* Equations (7) and (9) in the current study, and measured the *MET* in Figure 4. When plotting the *MET* Equation (9), the *BMI* was the mean of the 40 participants (23.1 kg/m^2^). We found that the *MET* in the current study was smaller than that of static pulling tasks. This was in contrast to that in the literature [54]. In a static working posture, fast-twitch fibers are mostly used and this type of muscle fiber has a low resistance to fatigue [54]. Static force exertion tasks may result in much faster muscle fatigue accumulation. In dynamic working, slow-twitch fibers are mainly used and these types of muscle fibers have a high resistance to fatigue [54]. Meanwhile, blood circulation during dynamic motions is better than in a static working posture [54]. Therefore, dynamic force exertion tasks may result in much slower muscle fatigue accumulation. However, the *MET* in the current study was smaller than that of the static pulling tasks. This may be attributed to the following reasons. In the current study, the participants grabbed the handle and pulled and walked on a treadmill. The only difference between the static pulling task in the literature [16,17,18,19,20,21,22] and our study was that the participants in our study needed to walk. The participants might need more effort to pull and walk than those in the static pulling tasks.

As shown in Table 2, the *MET* Equation (7) has lower *MADs* than Equation (9). When the *f_MVC_* is between 0.1 and 0.3, the curves of these two equations largely overlap (see Figure 4). When the *f_MVC_* is larger than 0.3, Equation (9) has a lower *MET*. The *f_MVC_* in the current study was between 0.09–0.27, which is less than 0.3. Therefore, further discussion is needed to select the best equation.

**Figure 5:**
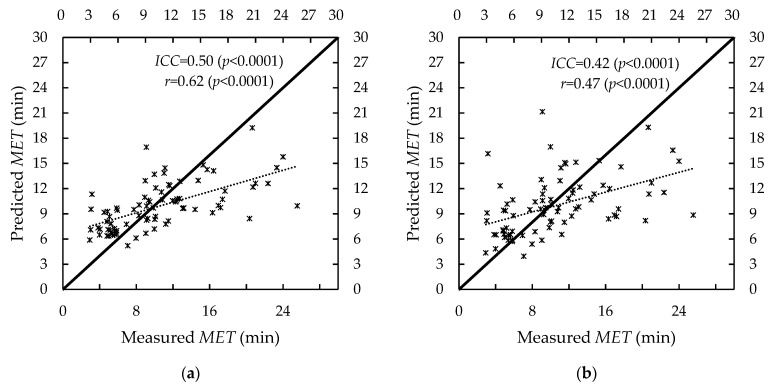
Comparison of measured and predicted *MET*. (**a**) Equation (7), (**b**) Equation (9).

In the literature [33,39,55], intraclass correlation efficients (*ICC)* and Pearson correlation coefficients (*r)* were usually calculated to describe the similarity and the relationship between the two data sets, respectively. We compared the measured and predicted *MET* for Equations (7) and (9) (see Figure 5) by calculating the *ICC* and *r*. These two statistics for Equation (7) were 0.50 (*p* < 0.0001) and 0.62 (*p* < 0.0001), respectively. The *ICC* and *r* for Equation (9) were 0.42 (*p* < 0.0001) and 0.47 (*p* < 0.0001), respectively. The *MET* Equation (7) has higher similarity and correlation. Therefore, the *MET* Equation (7) was more appropriate than Equation (9) in our pulling tasks.

### 3.2. Muscle Fatigue Recovery for Pulling Tasks

In Table 2, we found that *PS_i_* (*i* = 0, 1, …, and 6) increased with the time period. We constructed a muscle fatigue recovery model for pulling tasks based on the model proposed by Ma [30]:

(10) $$PS=PS_{0}+(MVC-PS_{0})(1-e^{-RR\times t})$$

*PS* (N) is a pull force at time *t*. *PS*_0_ (N) is the pull force at time 0 min. *RR* is the muscle fatigue recovery rate and min^−1^. *t* is time in min.

By logarithmic transformation of Equation (10), we have:

(11) $$RR\times t=\ln(\frac{MVC-PS_{0}}{MVC-PS})$$

If, $y=\ln\left(\frac{MVC-PS_{0}}{MVC-PS}\right)$, then, Equation (11) can be written as: y = *RR* × *t*.

By substituting data group A into y = *RR* × *t* and performing the simple intercept-free linear regression, we have:

y = 0.17*t*      (*R*^2^ = 0.80, *p* < 0.0001, *F* = 1134.67) (12)

That is, *RR* = 0.17. Therefore, the muscle fatigue recovery model for pulling tasks was:

(13) $$PS=PS_{0}+(MVC-PS_{0})(1-e^{-0.17\times t})$$

**Figure 6:**
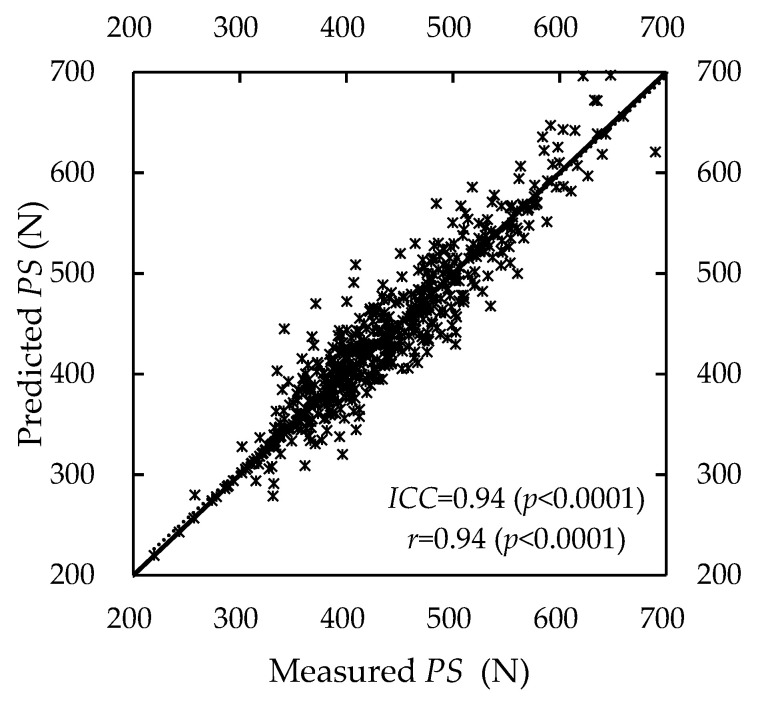
Measured and predicted *PS* showing *PS* recovery.

By substituting data groups A and B into Equation (13), we calculated the *MADs*. The *MADs* were 19.61 (±19.62) N and 20.23 (±17.71) N, respectively, for these groups. Both the *ICC* and *r* for the measured and predicted *PS* were 0.94 (*p* < 0.0001) (see Figure 6).

## 4. Discussion

The development and recovery of muscle fatigue for pulling tasks were studied in this paper. In the muscle fatigue test, an endurance experiment was conducted according to those experiments in the literature [30,31,33]. The muscle fatigue induction, that is, the decline of *MVC*, was 33.77 (±8.73)% *MVC*, which was between the 30 to 50% range reported in the literature [30,32,33,56].

### 4.1. Muscle Fatigue

In the muscle fatigue test, the *PS* decreases were (200.42 ± 64.59) N and (182.19 ± 62.70) N for 30 kg and 45 kg conditions, respectively. The *PS* decrease rate (*PS* decrease/*MET*) for the two load conditions was significantly different (*p* < 0.0001). The *PS* decrease rate for the 45 kg condition (30.83 ± 16.49) N/min was significantly higher (*p* < 0.05) than that of the 30 kg condition (15.35 ± 5.45) N/min. The load of the 45 kg condition was 1.38 times (106.6 N/77.2 N) greater than that of the 30 kg condition, while the *PS* decrease rate of the former was 2.01 times (30.83 N/min/15.35 N/min) greater than that of the latter. In Tang’s research [27], the load of the 40 kg condition was 1.2 times (122.99/102.9) greater than that of the 30 kg condition, while the *PS* decrease rate of the former was 1.53 times (27.44/17.93) greater than that of the latter. In these two experiments, both the *PS* decrease rates were faster than the load increase rates. Therefore, when the load increase is large, a longer rest time needs to be arranged.

As shown in Figure 4, the *MET* had a rapid decrease in the *f_MVC_* range of 0.1–0.2. This was consistent with these *MET* models in the static force exertion tasks [21,39,49,50] and those *MET* models in the dynamic forceful tasks [27,45,50,51]. When fitting the data in the current study, Equation (7) has a lower *MAD* and better similarity and correlation (see Figure 5 and Table 2). The *MADs* of Equation (7), 3.28 (±2.99) min for data group A and 3.22 (±2.88) min for data group B, were larger than those in the literature [27,33]. Those *MADs*, however, were lower than that in the backpacking tasks [45]. By comparing the *MADs* in the literature (see Table 3), we found that low *METs* has lower *MADs* [27,33], and a higher *MET* has a larger *MAD* [45]. The higher the *MET*, the higher the *MAD* is. When the participants performed the pulling tasks in the current study, they were required to keep their eyes forward and not allowed to chat or listen to music. Instead of being physically exhausted, the participants might have given up pulling because of boredom [45] when they walked with a load on the treadmill at a certain speed for a long time. This may be the reason for the *MADs* larger than 3 min in the current study.

The *f_MVC_* in the current study was between 0.09 and 0.27, while the *f_MVC_* in Tang et al. [27] was between 0.31 and 0.51. The *f_MVC_* values are outside each other’s range. We tried to reconstruct the *MET* model using the data from the current study and that of Tang et al. [27]. For this new data set, the *f_MVC_* was between 0.07 and 0.51. Two new *MET* equations can be obtained:

(14) $$MET=f_{MVC}^{-1.253}\;\;\;\;\;\;\;\;\;\;\;\;\;\;\;\;\;\;\;\;\;\;\;\;\;\;\;\;\;\;(R^{2}\;=\;0.96,\;p\;<\;0.0001)$$

(15) $$MET=e^{-3.218f_{MVC}}\times BMI^{0.885}\;\;\;\;\;\;(R^{2}\;=\;0.95,\;p\;<\;0.0001)$$

**Figure 7:**
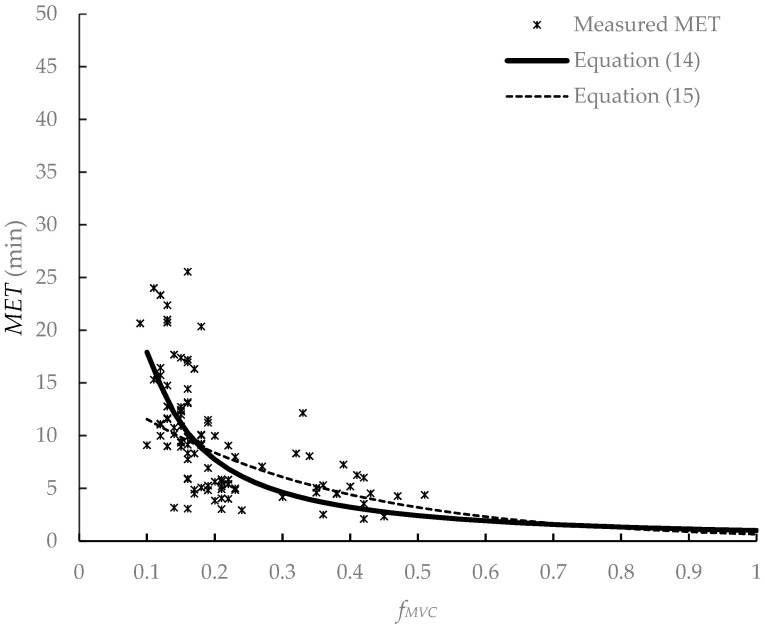
*MET* models for new data set. *f_MVC_* = forceful exertion/*MVC*.

The *MADs* for Equations (14) and (15) were (3.05 ± 2.76) min and (3.38 ± 3.26) min. The *ICC* and *r* values of Equations (14) and (15) were 0.62 (*p* < 0.0001) and 0.67 (*p* < 0.0001) and 0.43 (*p* < 0.0001) and 0.54 (*p* < 0.0001), respectively. As shown in Figure 7, Equation (14) fitted the new data better. By comparing Equations (7) and (14), we found the difference in the parameter was 0.008. Both equations were acceptable. In our pulling tasks, the *MET* models, according to static force exertion, better fit the data. Thus, the *MET* models in static pulling tasks [33] and dynamic pulling tasks in the current study have the same function. This verified our hypothesis that pulling tasks with a low walking velocity were a slow dynamic task. These results were similar to that of Hagberg [50]. This may be attributed to the static contractions of the upper body in static and dynamic pulling tasks. It has been reported that the predominance of sustained static muscular contractions in the muscles of the upper body is likely to be a source of fatigue and injury [57]. Bennett et al. [6] found that only the upper body muscles were considered for possible fatigue as they were involved in static contraction when they conducted forward pushing, forward unilateral pulling, and backward bilateral pulling tasks. Their forward unilateral pulling was similar to our pulling task in the current study.

### 4.2. Muscle Fatigue Recovery

The *PS* at times 0, 1, …, and 6 min were 72.7%, 75.9%, 78.7%, 81.6%, 84.16%, and 87.5% of the *MVC*. The increase in *PS* showed the recovery of muscle fatigue. After a rest time of 6 min, the *PS* recovered to 87.5% *MVC*. According to the 95% *MVC* recovery line recommended in the literature [48,58], the participants fully recovered after a rest time of 11.3 min. A rest time of about 12 min can be adopted to assign pulling tasks in the workplace to avoid muscle fatigue and reduce the risk of MSDs.

Based on Equation (13), we obtained the recovery of muscle force. Muscle force decrease (*MVC*-muscle force) has been used to quantify the progress of muscle fatigue in the literature [42,48,58]. Perceived force exertion and subjective assessment models have also been adopted to evaluate the forceful exertion level [59,60,61] or muscle fatigue level [35,36,37]. Following the literature [35,36,42,48,58], we defined the normalization of pull strength decrease (*NPSD*) using the following equation:

(16) $$NPSD=\frac{MVC-PS}{MVC}\times 100\%$$

*NPSD* was significantly positively correlated with *CR-10* (*r* = 0.63, *p* < 0.0001). The following equation was established to show the relationship between *CR-10* and *NPSD*:

(17) $$\text{CR-10}=20.143NPSD\;\;\;\;\;\;(R^{2}\;=\;0.88,\;p\;<\;0.0001)$$

Substituting Equations (13) and (16) into Equation (17), we obtained the following equation:

(18) $$\text{CR-10}=20.143\times e^{-0.17t}\times (1-\frac{PS_{0}}{MVC})$$

**Figure 8:**
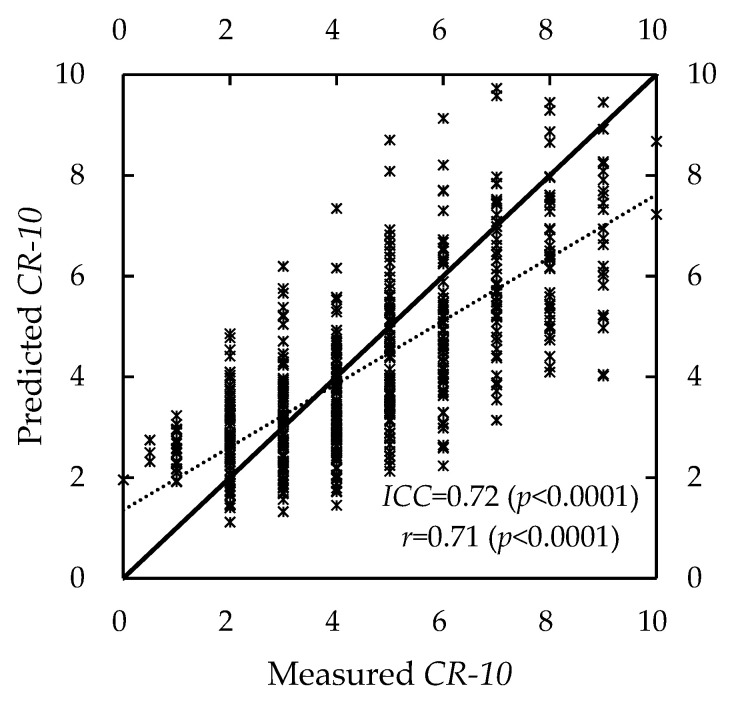
Comparison of predicted and measured *CR-10* using Equation (18).

The *MAD* of the predicted and measured *CR-10* was (1.27 ± 0.92). As shown in Figure 8, the *ICC* and *r* were 0.71 (*p* < 0.0001) and 0.72 (*p* < 0.0001), respectively. This *CR-10* assessment model was acceptable. It allows predictions of *CR-10* using *t*, *PS*_0_, and *MVC*.

### 4.3. Limitations

There are limitations to this study. Firstly, the pulling tasks were conducted on a treadmill and our participants walked at 1 km/h under our loading conditions, not any faster. In real pulling tasks, the operator may walk slower or faster depending on the load. The muscle fatigue and recovery models of the pulling and walking tests under different walking speeds will be interesting topics in the future. Secondly, all participants were young male adults. Older workers may have larger endurance time and female workers may have lower pull force. Our results, therefore, could be applied only to young male workers. Finally, only the *MET*, *PS*, and *CR-10* were analyzed in this study. Heart rate and EMG are also the appropriate physiological parameters for indicating physiological strain for pulling and walking tasks. Incorporating these physiological parameters into the *MET* and *CR-10* models will also be interesting topics in the future.

## 5. Conclusions

A muscle fatigue test and a muscle fatigue recovery test were conducted to explore the development and recovery of muscle fatigue for pulling tasks. In the muscle fatigue test, the operator pulled and walked on a treadmill with a velocity of 1 km/h and with loads of 30 or 45 kg. In the muscle fatigue recovery test, the operator had a rest and then measured their *PS* at the recovery time of 0, 1, 2, …, and 6 min. Load significantly affected the development of muscle fatigue for pulling tasks. The recovery time significantly affected the recovery of muscle fatigue for pulling tasks. Power function *MET* models were established to describe the development of muscle fatigue. A *PS* model and a *CR-10* model were both constructed to assess the recovery of muscle fatigue. Those models may be used to determine the work/rest allowance for pulling tasks under conditions similar to this study.

## Figures and Tables

**Table 1:** *PS* and *CR-10* value at recovery time for pulling tasks.

*t* (min)	*PS* (N)	*CR-10*
0	370.3 ± 69.2 ^A^	7.9 ± 0.9 ^a^
1	408.3 ± 68.7 ^B^	6.1 ± 1.2 ^b^
2	426.2 ± 66.4 ^BC^	5.0 ± 1.2 ^c^
3	442.1 ± 68.6 ^CD^	4.2 ± 1.4 ^d^
4	458.0 ± 73.1 ^DE^	3.5 ± 1.4 ^e^
5	472.1 ± 73.0 ^EF^	3.1 ± 1.4 ^f^
6	491.4 ± 74.3 ^F^	2.7 ± 1.2 ^g^

Note: The superscript letters indicate that the values of *PS* and *CR-10* are significantly different between different t, respectively, at α = 0.05.

**Table 2:** *MAD* values of the predictive models.

Models			*MAD* (min)
Body pull model [53]	Manenica		5.47 (±2.70)
Dynamic pull model [27]	Tang et al.		4.82 (±3.42)
Static pull model [19]	Exponential model		11.67 (±4.22)
	Power model		50.52 (±32.07)
Current study	Equation (7)	Group A data	3.28 (±2.99)
		Group B data	3.22 (±2.88)
	Equation (9)	Group A data	3.57 (±3.08)
		Group B data	3.70 (±3.74)

**Table 3:** The mean *METs* and mean *MADs* in the literature and in the current study.

	Mean *MET* (min)	Mean *MAD* (min)
Tang et al. [27]	4.40	0.95
Yi et al. [33]	4.93	1.32
Li et al. [45]	67.30	29.70
Current Study	10.50	3.25

## Data Availability

Data are available upon request.
